# Electrospun Combination of Peppermint Oil and Copper Sulphate with Conducive Physico-Chemical properties for Wound Dressing Applications

**DOI:** 10.3390/polym11040586

**Published:** 2019-04-01

**Authors:** Saravana Kumar Jaganathan, Mohan Prasath Mani, Ahmad Zahran Md Khudzari

**Affiliations:** 1 Department for Management of Science and Technology Development, Ton Duc Thang University, Ho Chi Minh City, Vietnam; 2 Faculty of Applied Sciences, Ton Duc Thang University, Ho Chi Minh City, Vietnam; 3 IJNUTM Cardiovascular Engineering center, School of Biomedical Engineering and Health Sciences, Faculty of Engineering, Universiti Teknologi Malaysia, Skudai 81310, Malaysia; zahran.kl@utm.my; 4 School of Biomedical Engineering and Health Sciences, Faculty of Engineering, Universiti Teknologi Malaysia, Skudai 81310, Malaysia; mohanprasathutm@gmail.com

**Keywords:** polyurethane, PM/CuSO_4_, physico-chemical properties, biocompatibility, wound healing applications

## Abstract

The ultimate goal in tissue engineering is to fabricate a scaffold which could mimic the native tissue structure. In this work, the physicochemical and biocompatibility properties of electrospun composites based on polyurethane (PU) with added pepper mint (PM) oil and copper sulphate (CuSO_4_) were investigated. Field Emission Electron microscope (FESEM) study depicted the increase in mean fiber diameter for PU/PM and decrease in fiber diameter for PU/PM/CuSO_4_ compared to the pristine PU. Fourier transform infrared spectroscopy (FTIR) analysis revealed the formation of a hydrogen bond for the fabricated composites as identified by an alteration in PU peak intensity. Contact angle analysis presented the hydrophobic nature of pristine PU and PU/PM while the PU/PM/CuSO_4_ showed hydrophilic behavior. Atomic force microscopy (AFM) analysis revealed the increase in the surface roughness for the PU/PM while PU/PM/CuSO_4_ showed a decrease in surface roughness compared to the pristine PU. Blood compatibility studies showed improved blood clotting time and less toxic behavior for the developed composites than the pristine PU. Finally, the cell viability of the fabricated composite was higher than the pristine PU as indicated in the MTS assay. Hence, the fabricated wound dressing composite based on PU with added PM and CuSO_4_ rendered a better physicochemical and biocompatible nature, making it suitable for wound healing applications.

## 1. Introduction

The largest organ in the human body is skin which plays a critical role in homeostasis and inhibits microorganism invasion. A wound is an injury to the skin when the skin tissue gets damaged. An injury is caused due to trauma, burns, or other by other factors like foot ulcers due to diabetes. Once the skin gets affected, it should be treated immediately, otherwise it can cause acute pain, serious infections, and also wound burden [1]. The treatment for the damaged skin involves the use of wound dressing which acts as a skin barrier and aids wound healing [2]. An ideal wound dressing should be biocompatible, non-allergic, providing a moist environment, facilitates gas exchange, adsorbs exudates, and reduces pain and risk of infection [3]. Further, it should display good biocompatibility and also resemble the native ECM structure for supporting new tissue formation [1,4]. Now-a-days, the nanofibrous scaffold was reported to be widespread in the tissue engineering applications. 

Nanofibers were fabricated using various processing techniques such as emulsion freeze drying, self-assembly, and phase separation [1]. The nanofibers fabricated using the above techniques were used in several applications such as energy applications [5], treatment of water and environment [6], healthcare, and biomedical engineering [7,8]. However, the nanofibers produced using these techniques does not meet the requirements of the ideal wound dressing membrane because of their large diameters and low porosity [9]. In addition, the nanofibers based on the electrospinning technique were widely used in biomedical applications because of their large surface area to volume ratios and porous structure [10]. The small interconnected pores of the electrospun membranes do not help suitable cellular response and tissue ingrowth. However, the pore size can be tailor made by optimizing or tuning the electrospinning parameters such as voltage, flow rate and collector distance [11]. Tang et al. reported that the increase in flow rate resulted in an increase in fiber diameter [12]. Similarly, Zhang et al observed that the low voltage favors the smaller fiber diameter while the high voltage results in broader fiber diameter [13]. Further, Yuan et al. reported that the thin fiber was formed for a larger collector distance and vice versa [14]. Tarus et al reported that the solvent with lower surface tension causes smoother fibers and fibers will be higher for other solvents with higher surface tension [15]. Further, electrospinning is a cost effective, facile, and well established technique used to fabricate nanofibers from both natural and synthetic polymers [16]. Some of the natural polymers used in the wound dressing were chitosan, collagen, alginate, gelatin, chitin, and silk fibroin. While the synthetic polymers used were poly(vinyl alcohol), poly(lactic acid), polycaprolactone (PCL), and poly(ethylene oxide) [1]. In this study, the Tecoflex EG 80A was used to fabricate the wound dressing scaffold. It belongs to the family of aliphatic polyether polyurethanes. It possesses good mechanical and ultraviolet (UV) stability [17]. For the past few decades, it was widely used in biomedical applications because of its biocompatible and biodegradable behaviour, oxidative and thermal stability [18,19].

An ideal scaffold for wound dressing must possess improved biocompatibility for influencing the fibroblast adhesion and also have antimicrobial activity to inhibit the microbes penetration. Unnithan et al. studied electrospun polyurethane (Mw = 110,000, Medical grade) scaffold with added emu oil fibers. It was shown that the emu oil incorporated PU scaffolds showed enhanced adhesion of fibroblast cells compared to the pristine polyurethane [20]. In another study, Jaganathan et al. fabricated polyurethane (Tecoflex EG 80A) scaffold added with grape seed oil, honey, and propolis. It was reported that the addition of grape seed improved the fibroblast adhesion compared to the pristine PU [21]. Hence these studies have proved that the addition of essential oil influenced the fibroblast adhesion and proliferation. Hence, these types of studies motivated us to use pepper mint (PM) oil for wound dressing applications. Mentha piperita L., known as peppermint oil, is a medicinal plant belongs to the Lamiaceae family [22]. It is widely found in temperate areas of the world, mainly in North America, Europe, and North Africa. Peppermint is composed of 29–48% menthol, about 20–31% menthone, 6.8% menthofuran, and 3–10% menthyl acetate. Other bioactive compounds include flavonoids, caffeic acid, polyphenols, tocopherols, carotenes, tannins, betaine, and choline [23,24,25]. Owing to their various bioactive constituents, it was used in food, cosmetic, and clinical applications. It has been utilized for treating colds, cancers, cramps, indigestion, sore throat, nausea, and toothaches. The polyphenolic constituents of PM oil might stimulate the immune system and help in preventing colds and similar viral infections [26]. These constituents also help in protecting the cells from free radicals and exert anticancer activity [27]. Further, it has been reported to possess some biomedical activities of an antibacterial, antiviral, and antioxidant nature [22]. Furthermore for wound dressing applications, it must impart or enhance the antimicrobial activity. Hence, it is necessary to add some efficient antibacterial agents into the fabricated scaffolds. In this research the copper sulphate (CuSO_4_) is selected. Literature reported that silver, copper, and zinc are less toxic with excellent antimicrobial activity compared to the other metallic particles. In addition to promising antimicrobial activity, copper also plays a vital role in aiding the various physiological and metabolic processes like promoting endothelial cells growth, accelerating angiogenesis, and equilibrium of extracellular skin proteins [28]. The aim of this research is to develop and characterize a novel wound dressing scaffold based on polyurethane added with PM oil and CuSO_4_.

## 2. Experimental

### 2.1. Materials

PU (Medical grade Tecoflex EG 80A) was supplied by Lubrizol, Wickliffe, OH, USA. PM oil was purchased from AEON, Johor, Malaysia. Copper sulphate (CuSO_4.5_H_2_O) was supplied by Sigma-Aldrich, Gillingham, UK. The solvent for PU is Dimethylformamide (DMF) which was obtained from Merck, Burlington, NJ, USA. The reagents utilized in clotting study was obtained from Diagnostic Enterprise, Thiruvananthapuram, India.

### 2.2. Preparation of PU, Composite Solution and Electrospinning process 

The homogeneous solution of PU, PM oil, and CuSO_4_ was prepared at 9 wt %, 4 v/v % and 4 wt % respectively. To make 9 wt % of PU solution, 0.315 g of PU pellets was added to 3.5 mL of DMF and stirred for 12 h at room temperature. Similarly, 80 µL of PM oil was added to 2 mL of DMF and stirred for 1 hr maximum to make 4 v/v % PM solution. Further, 4 wt % of CuSO_4_ solution was done by adding 0.080 g of CuSO_4_ in 2 mL of DMF and stirred for 2 h respectively. The prepared PM and CuSO_4_ homogeneous solution were added to the PU solution at an 8:1 v/v % for PU/PM and 8:0.5:0.5 v/v % for PU/PM/CuSO_4_. In this research, the electrospinning technique was used to develop a fibrous scaffold from the prepared homogenous solution. They were electrospun at a voltage of 11 kV, a flow rate of 0.3 ml/h, and a distance of 20 cm respectively [21]. The parameters were kept constant for all solutions for even comparison. The attained fibers were collected using aluminum foil and vacuum dried to remove any residues.

### 2.3. Morphological and Structural Characterization

Field emission scanning electron microscopy (FESEM) unit (Hitachi SU8020, Tokyo, Japan) was employed to analyze the morphology of as-spun membranes. The samples were coated with gold and imaged at different magnifications. The fiber diameter was measured using Image J software by choosing 30 locations randomly from the captured image. The wettability of the fibrous membranes was determined through static video contact angle equipment (AST products, Inc., Billerica, MA, USA). Sample with a size of 1 × 1 cm^2^ was placed on the measuring surface and deposited with a water droplet (0.5 µL). The image of the water droplet was captured within a few seconds via video camera. The computer integrated software measure the manual contact angle and the experiment was repeated for three trials. For IR analysis, the electrospun samples were measured over wavelengths between 600 and 4000 cm^−1^ at 4 cm^−1^ resolution in Nicolet iS 5, Thermo Fischer Scientific, Waltham, MA, USA. The ATR crystal employed is Zinc Selenium (ZnSe). The thermal stability was measured in thermogravimetric analysis (TGA) unit (PerkinElmer, Waltham, MA, USA) by heating a small piece of electrospun membrane (3 mg) between 30–1000 degree celcius under the nitrogen atmosphere. The remaining weight residue was measured at each temperature point and the obtained values were exported in an excel sheet. Finally, the surface morphology was determined using atomic force microscopy (AFM) unit (NanoWizard®, JPK Instruments, Berlin, Germany) under normal atmosphere by scanning the electrospun membrane in size of 20 um × 20 µm area and images in 256 resolution were obtained. Ra was calculated at three different locations using the integrated JPKSPM software.

### 2.4. Mechanical Characterization

Mechanical properties of the electrospun PU, PU/PM, and PU/PM/CuSO_4_ composite was measured by universal testing equipment (Gotech Testing Machines, AI-3000). The samples with a size of 40 mm × 15 mm was clamped in the testing machine with a gauge length of 20 mm. The test was performed at a velocity of 10 mm/min with a load cell of 500 N at room temperature. The computer integrated software displays the stress–strain curve from which the average tensile strength at maximum stress was measured. 

### 2.5. Coagulation Assays

#### 2.5.1. Activated Partial Thromboplastin Time (APTT) and Prothrombin Time (PT) Assay

PU, PU/PM, and PU/PM/CuSO_4_ scaffolds were cut into a size of 1 × 1 cm^2^ and incubated with 50 µl of platelet poor plasma (PPP) for 1 min at 37 °C. Afterwards, 50 µl of rabbit brain cephaloplastin reagent was further added and incubated for 2 min at 37°C. Finally, the blood clot was initiated by adding 50 µL of CaCl_2_ and the formation of the clot was investigated with a sterile steel needle. The time taken for the blood clot was noted using a chronometer. Similarly for PT assay, the samples size of 1 × 1 cm^2^ was incubated with 50 µl of obtained PPP for 1 min at 37 °C. After, it was added with 50 µL of NaCl–thromboplastin reagent (Factor III) which initiate the blood clot. The time taken for the clot formation was measured by chronometer.

#### 2.5.2. Hemolysis Assay

Hemolysis assay was done to investigate the toxicity of electrospun membranes with red blood cells. Initially, the samples (1 × 1 cm^2^) were soaked in 0.9% w/v of physiological saline at 37 °C for 30 min. Next, they were added with a mixture of aliquots of citrated blood and diluted saline (4:5) for 1 h at 37 °C. After, the samples were retrieved and the mixture was centrifuged at 3000 rpm for 15 min. Finally, the supernatant was aspirated and the optical density was recorded at 542 nm which depicts the release of haemoglobin. The percentage of hemolysis or hemolytic index was calculated using the formula 21:

Hemolysis ratio (HR)=(TS−NC)/(PC−NC)×100(1)

where TS, NC, and PC are measured absorbance values of the test sample, negative control, and positive control at 542 nm, respectively.

### 2.6. Characterization of In Vitro Biocompatibility

HDF (Human Skin Fibroblast Cells 1184, ECACC, UK, primary cells obtained from the dermis of the skin) were cultured in DMEM medium supplied with 10% fetal bovine serum and incubated at 37 °C with 5% CO_2_. The medium was replaced every 3 days. The electrospun membranes with dimensions of 0.5 cm × 0.5 cm were cut and placed in the 96 well plates. Once the cells reached 80% confluence, they were seeded onto the scaffolds at a density of 10 × 10^3^ cells/ cm^2^. MTS assay was used to determine the cell viability of the cells after 5 days culturing. After 5 days of culture, the medium in which cell is grown on the electrospun membranes was added with 20% of MTS solution and incubated for 4 h. After 4 hr, the absorbance was measured at a wavelength of 490 nm using a spectrophotometric plate reader.

### 2.7. Statistical Analysis

One way ANOVA followed by Dunnett post hoc test was performed for experiments results with three trails to determine statistical significance. The analyzed data were expressed as mean ± SD for three trials. A representative of three images was presented for qualitative experiments.

## 3. Result and discussion

### 3.1. FESEM Investigation

**Figure 1:**
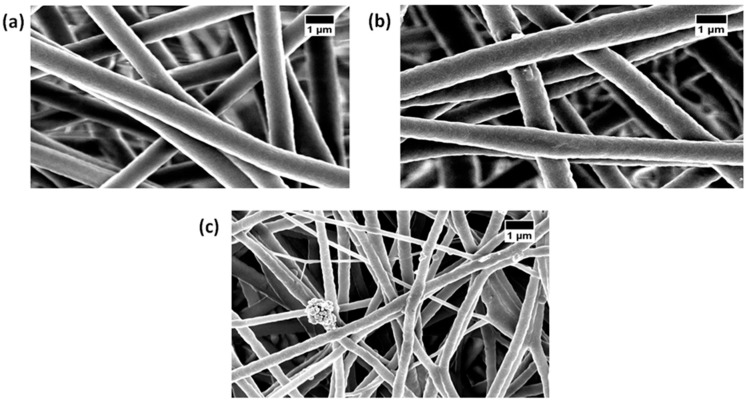
Field Emission Electron microscope (FESEM) images of (**a**) polyurethane (PU), (**b**) PU with added pepper mint (PU/PM), and (**c**) PU/PM/CuSO_4._

FESEM images of the prepared electrospun fibrous mats are shown in Figure 1. All the as-spun nanofibrous scaffolds showed a non-woven structure with smooth and beadless morphology. The average fiber size for pure PU was found to be 915 ± 137 nm, while the PU incorporated with PM and PM/ CuSO4 exhibited fiber sizes of 997 ± 134 nm and 359 ± 166 nm, respectively (mean differences were significant compared with pure PU (p < 0.05)). Hence, the addition of PM increased the fiber diameter of the pristine PU, while adding CuSO_4_ resulted in a decrease in fiber diameter. The alteration in fiber diameter morphology was due to a change in solution parameters while adding PM and CuSO_4_ into the polyurethane matrix. The increase in fiber diameter of PU adding PM oil was due to the interaction of PM oil bioactive constituents with the PU molecules. On the other hand, adding CuSO_4_ to the PU/PM, CuSO_4_ plays a vital role in reducing the fiber diameter which may be due to the interaction of PM oil bioactive constituents with the CuSO_4_ particles. Similar observations were seen in a recent work (Jaganathan et al.), which fabricated polyurethane (Tecoflex EG 80A) scaffold with added copper particles for wound dressing applications. It was found that the addition of copper particles resulted in the reduction of the fiber diameter which correlates with our observation. Further, in their work, the developed composites showed an enhanced proliferation rate of fibroblast cells than the pristine PU [29]. The fiber diameter of the fabricated composites showed a reduced fiber diameter compared to the pristine PU which might be conducive for the wound dressing applications. Further, EDS study was performed to confirm the presence of copper in the polyurethane matrix. From Table 1, it was evident that the polyurethane matrix showed some content of copper (1.5%) in addition to the carbon and oxygen content. 

### 3.2. FTIR Analysis

**Figure 2:**
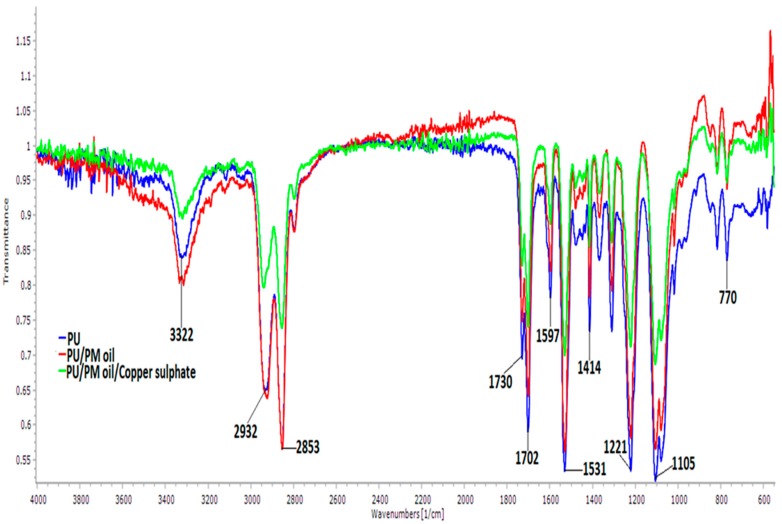
Fourier transform infrared spectroscopy (FTIR) of PU, PU/PM, and PU/PM/CuSO_4._

To evaluate the chemical composition of the developed fibrous membrane, FTIR spectra were inspected in wavelength range of 600–4000 cm^−1^ as indicated in Figure 2. The typical peaks present in the pristine PU are shown in Table 2.

The band at 3322 cm^−1^ indicates the stretching of NH group, peaks at 2932 and 2853 cm^−1^ represent the asymmetric and symmetric stretching of CH, and twin peaks at 1730 and 1702 cm^−1^ were attributed to the CO group. The vibration of the NH group was observed at 1531 and 1597 cm^−1^ and for CH vibrations it was shown at 1414 cm^−1^. The other sharp peaks at 1220, 1078, and 770 cm^−1^ indicate the CO group with respect to alcohol [21]. For composite membranes, it was found that no new peaks were observed but the intensity of PU was altered (increasing for PU/PM and decreasing for PU/PM/CuSO_4_) indicating a formation of a stronger hydrogen bond [20]. The hydrogen bond was formed between molecules of PM and CuSO_4_ with PU. The hydrogen bond formation was due to the combination of OH and CH molecules present in PM oil with the molecules of the pristine PU. Hence, the FTIR study confirms the existence of PM and CuSO_4_ in the polyurethane matrix.

### 3.3. Wettability Measurements

The contact angle is a measure of the wettability of the developed membranes. The static contact angle (CA) measurements of the pure PU and their composites were presented. The pure PU fibrous membrane showed a contact angle of 106° ± 3° indicating its hydrophobic behavior. Upon the incorporation of PM into the polyurethane matrix, the contact angle was increased to 111° ± 2°, indicating higher hydrophobicity than the pristine PU. On another hand, the addition of CuSO_4_ to the PU/PM resulted in the hydrophilic nature showing a contact angle of 82° ± 1°. Hence, the addition of CuSO_4_ improved the wettability of the polymer matrix. Jaganathan et al. electrospun polyurethane (Tecoflex EG 80A) scaffold incorporated with zinc particles for wound dressing applications. It was reported that the incorporation of zinc particles improved the wettability of the pristine PU which correlates with our observation. The contact of polyurethane was reported to be 100° ± 0.5774°, while polyurethane added with zinc nitrate displayed contact angle of 84° ± 4.041°. Further, the scaffold with improved wettability exhibited enhanced fibroblast cells adhesion than the pristine PU [30]. The addition of CuSO_4_ improved the wettability of the PU/PM which might be suitable for better fibroblast cell adhesion and proliferation. 

### 3.4. TGA Analysis

**Figure 3:**
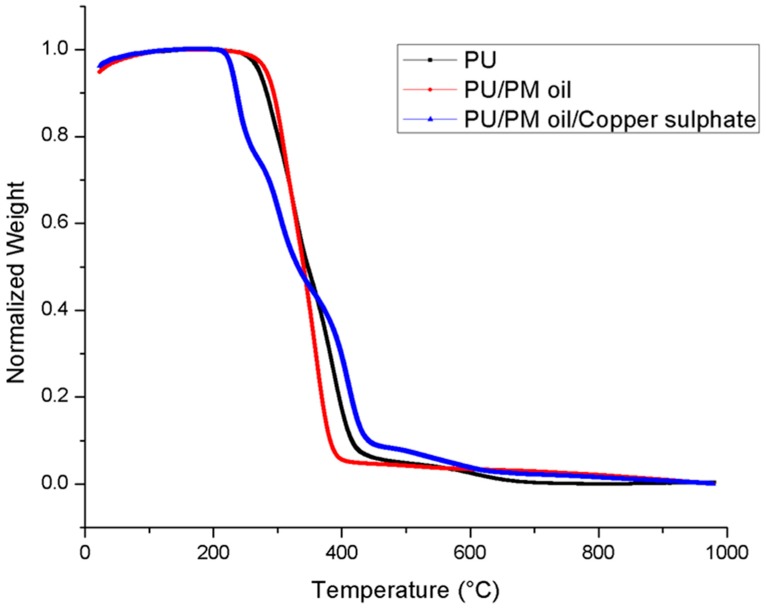
TGA of PU, PU/PM, and PU/PM/CuSO_4._

**Figure 4:**
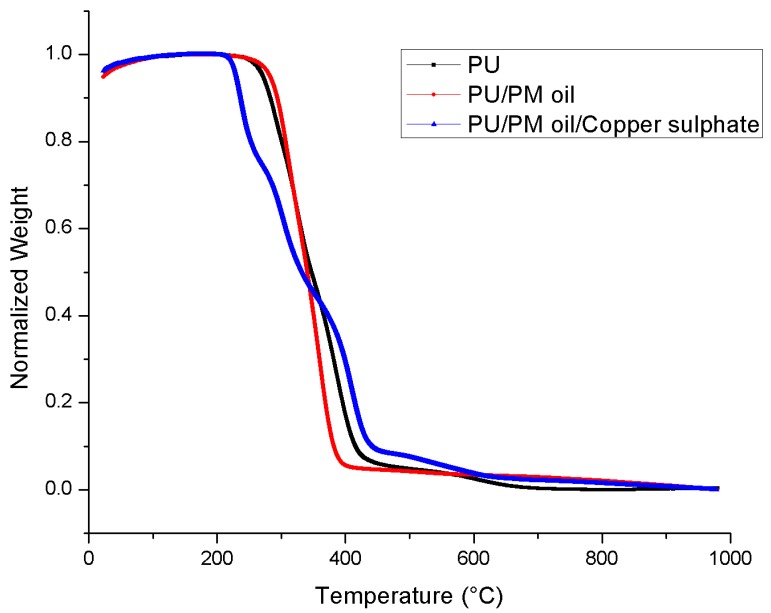
Weight residue of PU, PU/PM, and PU/PM/CuSO_4._

The thermal properties of electrospun PU, PU/PM, and PU/PM/CuSO_4_ are shown in Figure 3. In the case of pristine PU, the initial degradation temperature was observed to be 266 °C, while for electrospun PU/PM the temperature was increased to 286 °C and for PU/PM/CuSO_4_, it was decreased to 227 °C. Hence, the thermal properties were increased with the addition of PM. However, adding CuSO_4_ into the polymer matrix decreased its thermal stability. The decrease in the thermal stability was due to the moisture evaporation present in the copper sulphate pentahydrate. Further, DTG was performed for the electrospun membranes as indicated in Figure 4 and their weight loss peaks were listed in Table 3. From the results obtained, the electrospun PU/PM showed only three weight loss peaks compared to the pristine PU showing four weight loss peaks. However, electrospun PU/PM/CuSO4 showed weight loss peaks as that of pristine PU, but their weight loss intensity was decreased. Hence, the reduced weight loss peaks and decrease in weight loss intensity confirms the reduced weigh loss of the fabricated nanocomposites. Moreover, this attribute also confirms the presence of PM and CuSO4 in the polyurethane matrix.

### 3.5. AFM Analysis

**Figure 5:**
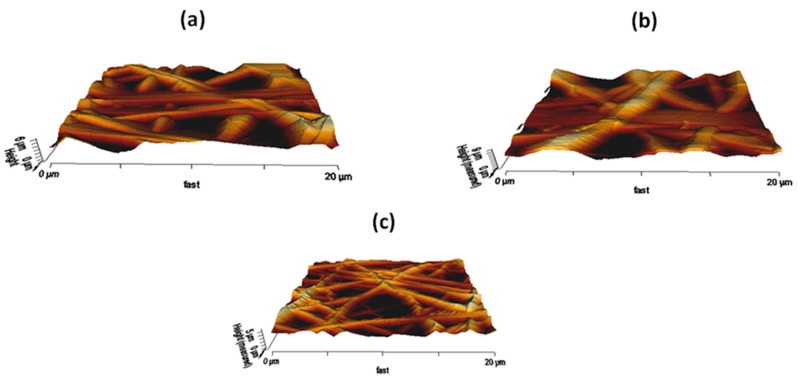
AFM images of (**a**) PU, (**b**) PU/PM, and (**c**) PU/PM/CuSO_4._

To analyse the effect of PM and CuSO_4_ incorporation on the surface roughness of the pristine PU, AFM was performed. The representative 3D images of the fibrous membranes are shown in Figure 5. The average roughness (R_a_) of the pristine PU ranged 776 ± 468 nm, while the PU incorporated with PM and PM/CuSO_4_ have an average roughness of 1039 ± 198 nm and 515 ± 123 nm, respectively. The surface roughness measurements depicted that the fabricated PU/PM composites have rougher surfaces, while PU/PM/CuSO_4_ had smoother surfaces compared to the pristine PU. Hyung Hwan Kim et al studied the effect of fiber diameter on surface roughness in electrospun polycaprolactone membrane. It was reported that the average surface roughness was increased with an increase in fiber diameter. Our electrospun PU/PM oil showed a larger fiber diameter which might favor the improvement in the surface roughness. In contrast, the electrospun PU/PM/CuSO_4_ displayed smaller fiber morphology which results in smooth surfaces [31]. Huag et al reported that the fibroblast cells prefer smoother surface to adhere and proliferate [32]. The smooth surface will have a smaller fiber diameter which would be suitable for the fibroblast cells to adhere and grow [18,31]. On other hand, Sharifi et al. reported that surface modified polycaprolactone fibrous membrane with increased surface roughness also favors the improved adhesion and growth of fibroblast cells [33]. The influence of surface roughness on the cellular response is still unclear and the various physico-chemical properties of the manufactured composites might have a role in the cell adhesion behavior. To state, the smaller fiber diameter favors the enhanced adhesion of protein which results in large cell attachment and proliferation [29]. Further, the adequate porosity also helps in efficient transport of nutrients and waste removal for better cell adhesion and growth [34].

### 3.6. Tensile Testing

**Figure 6:**
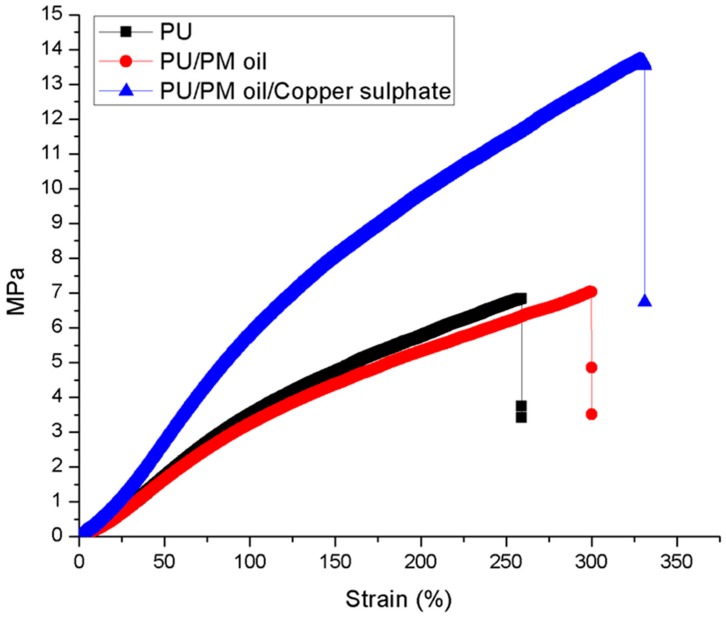
Tensile strength of PU, PU/PM, and PU/PM/CuSO_4._

Mechanical testing of the electrospun membranes was indicated in Figure 6 and Table 4. The tensile testing clearly indicated that the tensile strength of the pristine PU was enhanced on incorporating PM and CuSO_4_. PU showed a tensile strength of 6.83 MPa and, on adding PM and CuSO_4_, it was increased to 7.04 MPa and 13.60 MPa, respectively. 

The increase in tensile strength was perhaps due to the homogeneous dispersion of PM and CuSO_4_ in the polymer matrix. Unnithan et al. electrospun wound dressing scaffold utilizing polyurethane (Mw = 110,000), Medical grade) and emu oil. It was observed that the polyurethane/emu oil having smaller fiber diameter favored the enhancement of the tensile strength. They concluded that the addition of emu oil might favor the attachment of fibers owing to the hydrogen bond formation between polyurethane and emu oil molecules [20]. As reported earlier, FTIR analysis revealed the hydrogen bond formation in the fabricated composites which might have favored the enhancement of the tensile strength of PU/PM/CuSO_4_. Jaganathan et al. fabricated a wound dressing polyurethane (Tecoflex EG 80A) scaffold added with zinc nitrate. It was found that the zinc nitrate incorporation into the polyurethane exhibited improvement in the mechanical strength which correlates with our findings. They concluded this behavior was because of the smaller fiber diameter (568 ± 136.69 nm) of the electrospun composites compared to the polyurethane (1159 ± 147.48 nm) [30]. Our fiber diameter reduction was found to be similar to that reported above, which might have resulted in the improvement of the mechanical strength.

### 3.7. Blood Compatibility Measurements

**Figure 7:**
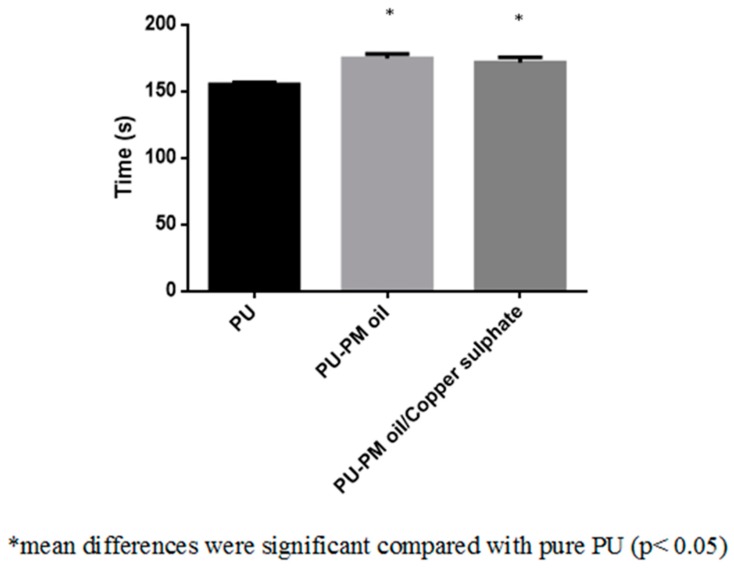
APTT assay of PU, PU/PM, and PU/PM/CuSO_4._

**Figure 8:**
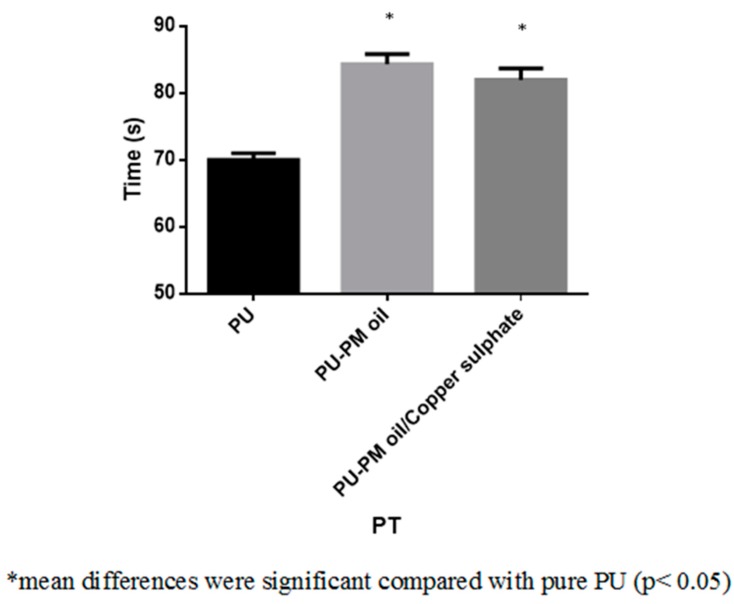
PT assay PU, PU/PM, and PU/PM/CuSO_4._

**Figure 9:**
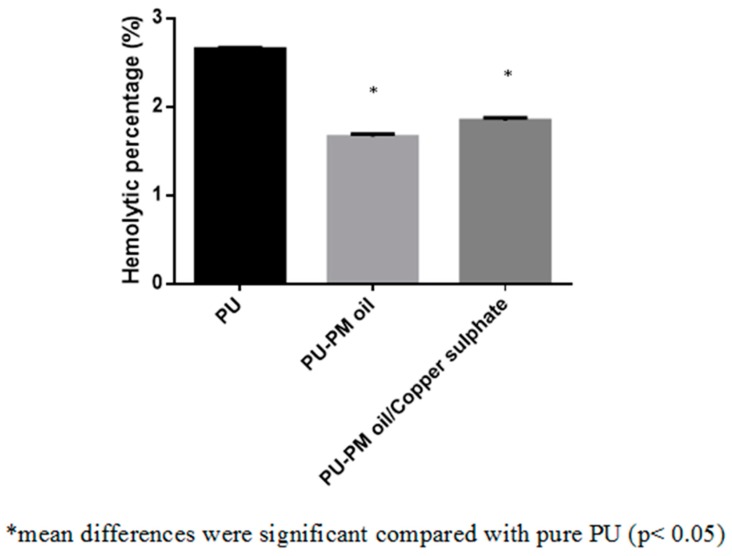
Hemolytic assay PU, PU/PM, and PU/PM/CuSO_4._

The dressing materials used for wound treatment will frequently come in contact with body fluids such as leakage of plasma and other blood components. If the dressing material does not have hemo-compatible properties, there might be risk of activating an undesired immune response which ultimately result in formation of thrombosis, inflammation, and foreign body reaction. This in turn delays the wound healing process. Hence, an ideal wound dressing should possess better blood compatible properties [35]. Blood clotting time of the electrospun PU, PM, and PM/CuSO_4_ was measured using APTT and PT assay. The coagulation assays showed the enhanced anticoagulant nature of the electrospun PU/PM and PU/PM/CuSO_4_ compared to the PU membrane as presented in Figure 7 and Figure 8. The developed PU/PM and PU/PM/CuSO_4_ mat exhibited blood clotting time of 175 ± 4 s and 172 ± 4 s (mean differences were significant compared with pure PU (p < 0.05)), while the PU showed blood clotting time of 155 ± 2 s as calculated in the APTT assay. Similarly, the developed PU/PM and PU/PM/CuSO_4_ mat exhibited blood clotting time of 84 ± 2 s and 82 ± 2 s (mean differences were significant compared with pure PU (p < 0.05)), while the PU showed blood clotting time of 70 ± 1 s as calculated in the PT assay. The existence of PM and CuSO_4_ in the PU matrix delayed the blood clotting time. Further, the hemolytic assay was measured to analyse the electrospun membranes toxicity with red blood cells. The results of hemolytic assay represent that the index value of the electrospun PU/PM and PU/PM/CuSO_4_ mat was found to be lower than pristine PU. The pristine PU showed an index value of 2.7% and for the electrospun PU/PM and PU/PM/CuSO_4_ mat, it was 1.66% and 1.85% (mean differences were significant compared with pure PU (p < 0.05)) as denoted in Figure 9. The fabricated composite was considered as non-hemolytic because its index value was less than 2% according to ASTMF756-00(2000) [21]. In blood compatibility assessments, the electrospun PU/PM oil showed prolonging blood clotting time compared to the pure polyurethane. However, there is a slight reduction in the blood clotting time while blending CuSO_4_ to the PU/PM but the observed blood clotting values were still higher than the pristine PU. According to Huang et al, the blood compatibility is influenced by multiple surface parameters such as wettability, fiber diameter, and surface roughness [36]. Jaganathan et al. electrospun a novel scaffold based on polyurethane and mustard oil. They found the improvement in the blood compatibility of the pristine PU with the addition of mustard oil and reported this might be because of an increase in surface roughness [37]. In another study, Ayyar et al. electrospun polyurethane scaffold incorporated with indhulekha oil and determined its anticoagulant nature. It was found that the electrospun PU/indhulekha oil composite with hydrophobic behavior showed prolonged blood clotting time than the pristine PU [38]. Further, Jaganathan et al. 2017 developed polyurethane scaffold loaded with castor oil using the electrospinning technique. The developed PU/castor oil showed prolonged blood clotting time compared to the pristine PU and this may be due to their smaller fiber diameter [39]. Hence, the addition of PM and CuSO4 into the PU matrix caused reduced fiber diameter morphology (PU/PM/CuSO_4_) and hydrophobic behavior (PU/PM) which might have resulted in the improvement of the anticoagulant nature.

### 3.8. Cytocompatibility Measurements

**Figure 10:**
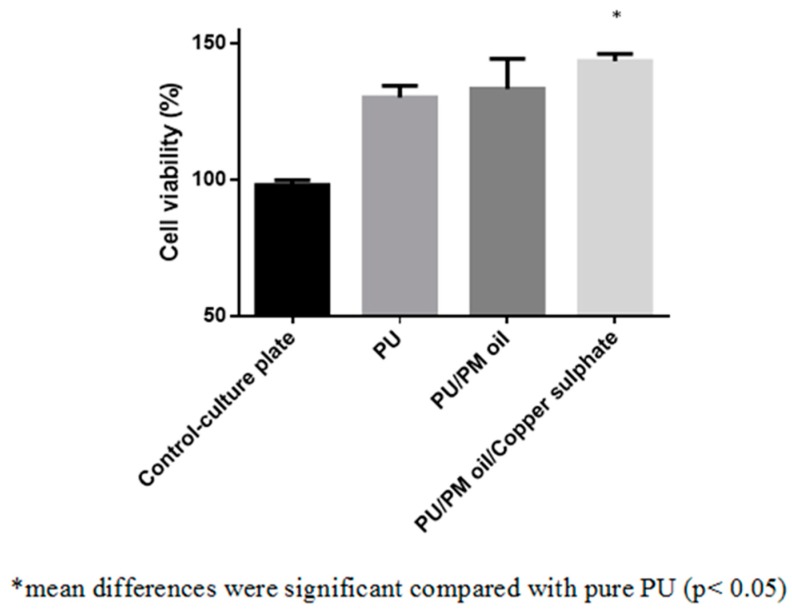
MTS assay PU, PU/PM, and PU/PM/CuSO_4._

Figure 10 presents the HDF cell viability in the electrospun membranes evaluated through MTS assay. After five days of cell culture, it was observed that the HDF cells were well adhered and proliferated in all electrospun membranes compared to the control plates. The cell viability percentage for the PU membrane was reported to be 130 ± 4%, while the electrospun PU/PM and PU/PM/CuSO_4_ composites showed cell viability of 133 ± 11% and 144 ± 3%, respectively. Further, HDF cells viability in the electrospun PU/PM/CuSO_4_ composites was observed to be higher than the PU/PM composites. As reported in the introduction section, PM oil contains polyphenols as one of their bioactive constituents. It has been reported that the phenolic components help in protecting the fibroblast cells against oxidative stress caused by hydrogen peroxide resulting in cell proliferation and migration. The phenolic compounds present in the PM oil might have influenced cell viability [40]. The reason for the improved HDF cell viability was perhaps due to their smaller fiber diameter (PU/PM) and hydrophilic behavior (PU/PM/ CuSO_4_) [41,42]. Hence, the improved cellular response of the developed composites might be suitable for wound dressing applications.

## 4. Conclusion

This work successfully evaluated the physicochemical and biocompatibility properties of electrospun composites based on polyurethane (PU) added with pepper mint (PM) oil and copper sulphate (CuSO_4_). The fabricated composites showed improved physicochemcial properties compared to the pristine PU. Blood compatibility studies showed improved anti-coagulation time and less toxic behavior for the developed composites than the pristine PU. Finally, the cell viability of the fabricated composite was higher than the pristine PU as indicated in MTS assay. Hence, the fabricated wound dressing composite based on PU/PM and PU/PM/CuSO_4_ rendering better physicochemical and biocompatible nature, mark its suitability for wound healing applications. In the future, it would be interesting to explore the cell proliferation and morphology characteristics of HDF cells using SEM analysis at different time points, which will further validate the claims of cytotoxicity.

## Figures and Tables

**Table 1:** EDS table of electrospun membranes.

Membrane	Carbon	Oxygen	Copper
PU	78.9 ± 0.8	21.1 ± 0.8	-
PU/PM	79.1 ± 1.2	20.9 ± 1.2	-
PU/PM/CuSO_4_	75.6 ± 0.3	23 ± 0.3	1.5 ± 0.2

**Table 2:** Functional groups in the electrospun PU membranes.

Peaks	Band name
3322 cm^−1^	N–H
2932 and 2853 cm^−1^	C–H
1730 and 1702 cm^−1^	C=O
1531 and 1597 cm^−1^	Vibrations of N–H
1414 cm^−1^	Vibrations of C–H
1221, 1105, and 770 cm^−1^	C–O corresponding to the alcohol group

**Table 3:** Weight loss peaks of electrospun PU, PU/PM, and PU/PM/CuSO_4._

S.NO	PU	PU/PM	PU/PM/CuSO_4_
First weight loss	210 °C to 302 °C	228 °C to 327 °C	206 °C to 270 °C
Second weight loss	302 °C to 353 °C	327 °C to 434 °C	270 °C to 353 °C
Third weight loss	353 °C to 494 °C	-	353 °C to 476 °C
Fourth weight loss	494 °C to 760 °C	-	476 °C to 664 °C

**Table 4:** Mechanical testing values of electrospun membranes.

Membrane	Tensile strength MPa	Elastic Modulus MPa	Elongation at break (%)
PU	6.83	3.51	259.16
PU/PM	7.04	2.95	300.10
PU/PM/CuSO_4_	13.60	5.37	331.15
